# Polarized recombination of acoustically transported carriers in GaAs nanowires

**DOI:** 10.1186/1556-276X-7-247

**Published:** 2012-05-14

**Authors:** Michael Möller, Alberto Hernández-Mínguez, Steffen Breuer, Carsten Pfüller, Oliver Brandt, Mauricio M de Lima, Andrés Cantarero, Lutz Geelhaar, Henning Riechert, Paulo V Santos

**Affiliations:** 1 Materials Science Institute, University of Valencia, Paterna 46980, Catedrático José Beltrán 2, Spain; 2, Paul-Drude-Institut für Festkörperelektronik, Hausvogteiplatz 5-7, Berlin 10117, Germany; 3, Fundació General de la Universitat de València, Valencia 46010, Spain

**Keywords:** Charge transport, Spin transport, GaAs, Nanowires, Surface acoustic waves, Photoluminescence, Polarization

## Abstract

The oscillating piezoelectric field of a surface acoustic wave (SAW) is employed to transport photoexcited electrons and holes in GaAs nanowires deposited on a SAW delay line on a LiNbO_3_ crystal. The carriers generated in the nanowire by a focused light spot are acoustically transferred to a second location where they recombine. We show that the recombination of the transported carriers occurs in a zinc blende section on top of the predominant wurtzite nanowire. This allows contactless control of the linear polarized emission by SAWs which is governed by the crystal structure. Additional polarization-resolved photoluminescence measurements were performed to investigate spin conservation during transport.

## Background

Semiconductor nanowires offer new perspectives for low-dimensional semiconductor devices since the small radius favors mesoscopic size effects and lifts the epitaxial constraints associated with the growth of dissimilar materials. In addition, the geometry of nanowires enables them to function as active device elements and interconnects, which can lead to highly integrated optoelectronic device structures. In this respect, the crystal structure of the nanowires is of importance since it controls the optoelectronic properties. Exploitation of these properties normally requires doping and contacting of non-planar, nanometer-sized structures for the application of electric control fields. Here, we applied surface acoustic waves (SAW) to remotely control the carrier recombination in GaAs/AlGaAs core-shell nanowires.

We have recently demonstrated that the oscillating piezoelectric field of a SAW can transport photoexcited charge carriers in GaAs nanowires as well as control the spatial location of exciton recombination along the nanowire axis [1]. In this contribution, we use polarized photoluminescence (PL) spectroscopy with spatial resolution to address the linear polarized emission characteristics of the photoexcited carriers along the nanowire. In addition, we face the question whether optically generated electron spins can be maintained during acoustic transport in nanowires.

## Methods

**Figure 1:**
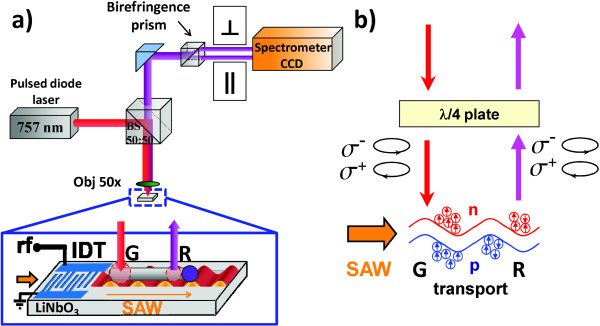
**Experimental setup.** (**a**) Experimental setup: the nanowire is excited by a tightly focused pulsed laser beam at the edge of the nanowire facing the interdigital transducer (excitation spot G) used for SAW generation. The PL signal emitted along the nanowire axis is split by a birefringence prism into two orthogonally polarized rays. The rays with polarization parallel (∥) and perpendicular (⊥) to the nanowire axis are detected on the upper and lower regions of the CCD images, respectively. (**b**) Schematic diagram of the spin transport measurements. Linear polarized light is converted into circularly polarized by a *λ*/4 plate, which allows to excite holes and electrons with one preferential spin polarization [spin-up (*σ*^+^) or spin-down (*σ*^−^)].

The nanowires used in our study were grown by molecular beam epitaxy (MBE) using a self-assisted vapor-liquid-solid growth process on Si(111) substrate [2]. They consist of an undoped GaAs core of 106±18 nm diameter coated with 22±9 nm Al_*x*_Ga_1−*x*_As shell with *x*=0.1. The nanowires, which have a predominant wurtzite structure, were then mechanically dispersed on the surface of a 128°-rotated Y-cut X-propagating LiNbO_3_ crystal containing two floating electrode unidirectional transducers designed to generate SAWs at multiple harmonics of the fundamental wavelength *λ*_SAW_=35*μ*m. The experiments were carried out using the second harmonic with *λ*_SAW_=17.5 *μ*m, which correspond to the SAW frequency and period of *f*_SAW_=226.5 MHz and *τ*_SAW_=4.4 ns, respectively. The experiments were performed on those single nanowires which were incidentally aligned with the SAW propagation direction (see Figure 1a).

The results shown here were obtained at low temperatures (20 K) by mounting the sample in a microscope cryostat for spatially resolved photoluminescence measurements (see Figure 1a). A 757-nm diode laser yielding 150 ps wide pulses was used as excitation source, the repetition rate of the laser being synchronized with the SAW frequency. The linearly polarized laser beam was focused onto the nanowire by using a 50× objective (spot diameter of approximately 1.5 *μ*m on the sample). The PL emission of the nanowire, collected by the same objective, was split by a birefringence prism into two orthogonally polarized rays and imaged on the entrance slit of a spectrometer. The rays with polarization parallel and perpendicular to the nanowire axis are then detected with spatial resolution (about 1 *μ*m) on the upper and lower regions of a cooled charge-coupled device camera (CCD), respectively. For the spin transport measurements, a *λ*/4 plate was put into the beam path before the objective in order to convert the linear polarized light into a left-handed circularly polarized one and vice versa (see Figure 1b).

## Results and discussion

**Figure 2:**
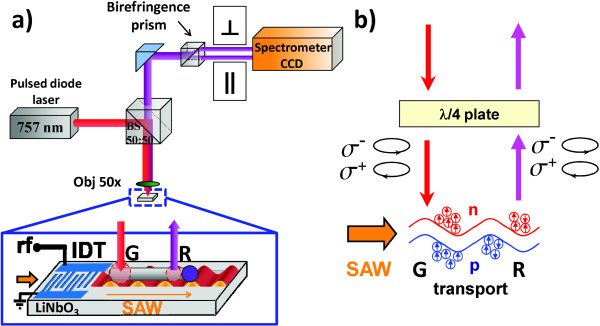
**Linear polarized PL emission with SAW off/SAW on.** Polarized PL with spatial resolution excited by a tightly focused laser beam close to the nanowire edge facing the acoustic transducer (position G, cf. diagrams on the left side). (**a**) In the absence of a SAW, the emission is restricted to the region close to the excitation spot and is polarized perpendicular to the nanowire axis. (**b**) Application of acoustic power of 12 dBm induces the transport of electrons and holes to a remote position R, where they recombine emitting light polarized mainly parallel to the nanowire axis. Spatial PL intensities along the nanowire axis integrated from 796.6 nm to 823.3 nm for the emission polarized perpendicular (blue circles) and parallel (red triangles) to the nanowire axis in the absence (**c**) (open symbols) and presence (**d**) (filled symbols) of a SAW.

Figure 2a,b displays the PL images excited by a tightly focused laser spot close to the end of the nanowire facing the acoustic transducer (cf. diagrams on the left of Figure 2) in the absence (Figure 2a) and presence (Figure 2b) of a SAW. In the upper and lower part of the images, the spectrally (horizontal scale) and spatially (vertical scale) resolved nanowire emission polarized parallel and perpendicular to the nanowire axis are represented respectively. The corresponding spatial PL profiles integrated from 796.6 to 823.3 nm are depicted in Figure 2c,d. Without acoustic power (Figure 2a), the emission is restricted to the region close to the excitation spot (position G in the diagrams on the left of Figure 2) and is enlarged due to diffusion processes along the nanowire. When a SAW is applied, the PL intensity at G reduces and a second emission at a remote point (position R) appears a few micrometers away from G along the SAW propagation direction. The reduction at G is due to the induced spatial separation of electrons and holes by the SAW piezoelectric field, which decreases the radiative recombination probability at G. The emission at R is attributed to the transport of the photoexcited electrons and holes along the nanowire axis and their recombination at the remote position, as we have recently demonstrated [1]. The slight PL emission appearing between G and R are associated to the recombination of some carriers at traps as stacking faults, impurities, or defects.

Without acoustic power, the broad emission band at G is highly polarized perpendicular to the nanowire axis (see Figure 2a,c). When a SAW is applied, the emission at G maintains its polarization behavior while the recombination at R is polarized parallel to the nanowire axis (see Figure 2b,d). The emission at G is consistent with the optical selection rules expected for the wurtzite GaAs nanowires [3] where the band edge emission is dipole allowed only if the electric dipole moment is perpendicular to the wurtzite *c*-axis [4]. The emission at R, however, does not coincide with these selection rules. Since the recombination of the transported charge carriers take place at a remote position the crystal structure of that region may be different. Indeed, the emission energy of approximately 818 nm - which is exactly the band gap energy of zinc blende bulk GaAs [5] - suggests that recombination takes place in a zinc blende region at the top of the nanowire, opposite to the acoustic transducer. This is supported by transmission electron microscopy measurements, which reveal a zinc blende section in the top region of the nanowires probably created during the cooling down after growth, as previously observed in CBE- and MBE-grown GaAs nanowires [6,7]. The emission polarized parallel to the nanowire axis at R is consistent with the emission characteristics observed in zinc blende GaAs nanowires [8,9]. Here, the emission is preferentially polarized along the nanowire axis due to the dielectric mismatch between the nanowire and its surroundings. The degree of linear polarization, *P*=(*I*_⊥_−*I*_∥_)/(*I*_⊥_ + *I*_∥_) - where *I*_⊥_ and *I*_∥_ are the PL polarization intensities detected perpendicular and parallel to the nanowire axis, respectively - at the excitation spot G, is approximately + 88*%*(perpendicular) in the absence of a SAW, whereas at the remote position R the polarization becomes equal to approximately −70*%*(parallel) with acoustic power.

**Figure 3:**
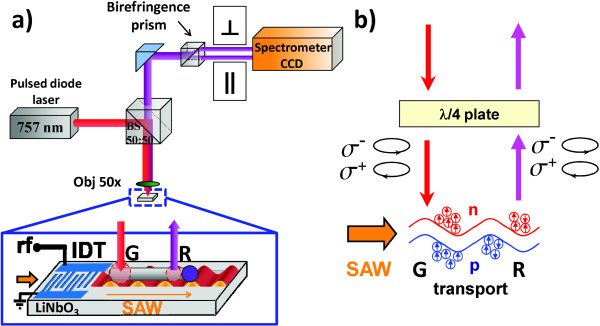
**Spin transport along nanowires.** Spatially resolved right-handed circularly polarized PL emission (*I*_+_) excited by a tightly focused, right-handed circularly polarized laser beam (*σ*^+^) in the absence (**a**) and presence (**b**) of a SAW. The PL intensity along the nanowire axis for incident right-handed (*σ*^+^, *solid line*) and left-handed (*σ*^−^, open circles) circularly polarized light integrated from 806 nm to 816 nm (blue lines and circles) and from 813 nm to 823 nm (red lines and circles) in the absence (**c**) and presence (**d**) of an acoustic power of 12 dBm. Spectra are arbitrarily shifted.

Similar polarization results have been obtained for all probed nanowires, independent of the polarization of the exciting laser. This also implies that the electron spins are not conserved during transport. To further support the last conclusion, we have carried out transport experiments where spin-up and spin-down electrons were generated on one extreme of the nanowire using right-handed (*σ*^+^) and left-handed (*σ*^−^) circularly polarized light, respectively. The circular polarization of the PL was detected with spatial resolution using *λ*/4 plate and a birefringence prism (see Figure 1). For simplicity, only the right-handed, circularly polarized emission (*I*_+_) is considered in the following discussion. For incident right-handed, circularly polarized light (*σ*^+^), the spatially resolved *I*_+_profiles in the absence and presence of a SAW are shown in Figure 3a,b, respectively. Figure 3c,d presents the *I*_+_ profiles along the nanowire axis integrated around the spectral positions 811 nm (blue) and 818 nm (red), in the absence and presence of a SAW for *σ*^+^ (lines) and *σ*^−^ (circles). Without acoustic power, the PL is emitted around 811 nm at the excitation spot G (Figure 3a). When a SAW is applied, a remote spot appears emitting at 818 nm (Figure 3b), as observed in the linear polarization measurements. From Figure 3c,d one sees that *I*_+_ is the same for *σ*^+^and *σ*^−^excitations (cf. solid lines with open circles) at G. This implies that the initially generated spin-up or spin-down electrons have lost their spin polarization in the wurtzite phase at G leading to evenly distributed spin states. The same result is observed for the recombination along the wire and at R where no difference of *I*_+_ of the transported electrons between *σ*^+^and *σ*^−^ can be observed.

## Conclusions

We have investigated the acoustic transport of charge along GaAs nanowires. We have shown that the transported electrons and holes recombine in zinc blende sections within the predominant wurtzite nanowire. In addition, spin polarization-resolved photoluminescence measurements have revealed that the spin polarization is lost during transport.

## Competing interests

The authors declare that they have no competing interests.

## Authors’ contributions

MM, AHM, and PVS performed the experiments on acoustic transport and analyzed the data. SB grew the NW sample and CP developed a process for harvesting NWs from as-grown ensembles and for dispersing them onto the LiNbO_3_ substrates. LG supervised NW growth and, together with OB, AC and HR, contributed to the analyses of the data and discussions. PVS conceived the experiments, supervised the project and, together with MM and MMLJ, prepared the manuscript. All authors read and approved the finalmanuscript.

## Authors’ information

MM is PhD student, MMLJ is senior researcher and AC is the director of Materials Science Institute of the University of Valencia. AHM, SB and CP are post-doc researchers. LG, OB and PVS are senior researchers and HR is the director of the Paul-Drude-Institut für Festkörperelektronik, Berlin.
